# Analysis of the Intelligent Tourism Route Planning Scheme Based on the Cluster Analysis Algorithm

**DOI:** 10.1155/2022/3310676

**Published:** 2022-06-28

**Authors:** Na Lou

**Affiliations:** Zhengzhou College of Finance and Economics, Zhengzhou, Henan 450000, China

## Abstract

In view of the problems of the traditional cluster analysis algorithm such as strong dependence on the initial cluster center, the traditional *k*-means cluster analysis algorithm is improved and the experiment proves that the improved algorithm has a better clustering effect; in view of the problems of the traditional tourism route planning, the improved *k*-means cluster analysis algorithm is applied to the intelligent tourism route planning scheme design and an intelligent tourism planning scheme based on the cluster analysis algorithm is proposed; the tourists' preference metric is fully considered, and the experimental results show that the scheme has certain reasonableness and reference value.

## 1. Current Situation of Tourism Route Planning

### 1.1. The Development of the Tourism Industry

With the improvement of the economy and the increase in people's happiness, China's tourism industry has made great achievements since the reform and opening up, both at home and abroad, China's tourism industry with concept features such as tourist cities and tourist hometowns has been deeply rooted in people's hearts [1]. In 2021, under the grip of the epidemic, China still received nearly 93 million people, while the total number of travel agencies in the country was more than 40,000, an increase of 4.3% year-on-year, indicating that under the epidemic, the number of people going to travel in China with good protection against the epidemic is still incessant, which also reflects the “vitality” of China's tourism industry under the epidemic from the side. The tourism industry in China is “vibrant” under the epidemic [2].

However, according to the “Analysis of Tourism Economy in 2021 and Development Forecast in 2022” released by China Tourism Research Institute, China's national tourism economy in 2022 is still suffering from a decline; in 2021, more than 70% of China's scenic spots suffered serious losses; a large part of this is due to not planning tourist routes, not understanding the needs of tourists, and other reasons [3]. However, there are still a lot of scenic spots that profited than in previous years because such scenic spots mastered the “flow” code, cracked the epidemic under the core of tourism as it was people-oriented, and mastered the wisdom of route planning, understanding the needs of tourists, and knowing what tourists want. Therefore, in today's development situation of the new crown epidemic, constructing intelligent tourism route planning is an important part of developing tourism.

### 1.2. Definition and Classification

Tourism planning, in academic circles, has three definitions, which are as follows [4].

The first one is the formulation and planning of tourism development goals and programs for a specific area in the future period of time, and then the tourism development and construction of that specific area after approval and adoption by relevant government departments or government agencies. In tourism planning, it is necessary to coordinate the overall situation and provide specific implementation plans and detailed guidance programs for the tourism practice of the region [5].

The second type is a tourism work plan based on the changing factors of the market, history, and current situation of the tourism industry. Under this plan, it is necessary to coordinate all available resources, such as urban and rural areas, so that the tourism industry in each region can develop in a coordinated and competitive way, and eventually achieve the effect of a hundred flowers [6].

The third type is an economic and technical act, i.e., intellectual resources, of which economy and technology are the primary conditions. The essence of this definition is the question of how to coordinate the relationship between the economy and the tourism industry, using the appropriate economic instruments and technical resources to determine the status and composition of the tourism industry in the national economic plan, and to propose goals for the development of the tourism industry, which, of course, is defined at the level of national economic development.

**Figure 1:**
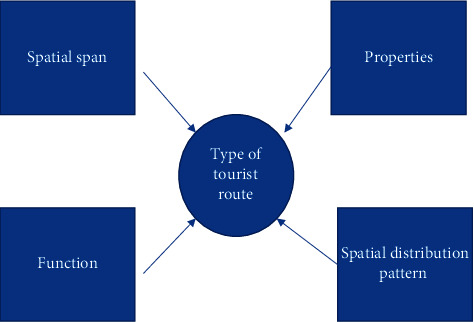
Classification of tourist routes.

In this paper, the study is focused on regional tourism planning, i.e., it corresponds to the first definition mentioned above. Among the types of tourism route planning, there are again the following [7] as shown in Figure 1.

Firstly, according to the spatial span, it can be divided into large- and medium-scale tourism routes and small-scale tourism routes. In the large- and medium-scale tourism routes, the travel agency needs to contact the source of tourists and a series of tourist paths of tourist places in advance; secondly, the tourism route on a small scale refers to the tour route of tourist attractions and the arrangement of related tour projects to meet the tourists' psychological and physiological needs, such that tourists can feel the joy and pleasure of tourism during the journey [8]. Secondly, according to the attribute, the tourist route can be divided into peripatetic and stay-type tourist routes. The circumambulation tourism route obviously refers to the global browsing and sightseeing, experiencing the whole city or the entire tourist attractions, such as the former residence of a historical figure, and so on, and then feeling the local history and culture and life; a stay-type tourism route is obviously a very short period of short-term sightseeing behavior; this is mainly for small scenic spots, and historical and cultural heritage is not deep enough in such scenic spots [9]. For this type of scenic spot, the use of stay-type tourism route planning is a good choice; after all, the tourism team needs to consider the overall travel experience; the inclusion of stay-type tourism routes can take into account the feelings of most people and points of interest, so as to not to leave behind classic scenic spots [10].

The third is divided by functional purpose, which can be divided into sightseeing, scientific research, adventure, thematic, shopping, and leisure vacation type. Sightseeing type is obviously for sightseeing scenery, including urban scenery, scenic scenery, rural scenery, etc.; scientific research type is mainly for some professionals' who are into the research and development of some scenic spots and set up; adventure type is mainly for people seeking excitement and adventure significance in some scenic spots. Thematic type is mainly for some specific topics, or different types of scenic areas; shopping type of tourism route planning has shopping as the main body, and the entire tourist route guides will guide tourists to spend; for leisure and vacation type, the main content is leisure and vacation, catering to people who are looking to relax from the usual work pressure [11].

### 1.3. Tourism Route Planning Research Status

After the above analysis, the economic growth trend of China's tourism industry in recent years has slowed down under the epidemic, but it is still optimistic on the whole. However, China's tourism industry still faces many challenges, and in order to solve various challenges, many research scholars based on tourism professions have put forward many insights [12]. For example, for specific regional tourism planning routes, Zhou Shengkang et al. took 26 attractions in Huang and Zhong Autonomous Prefecture as research objects and constructed a 16-indicator evaluation system for tourist attractions in four aspects: talent, system, facilities, and environment, while using qualitative and quantitative calculations to obtain the weights of each indicator, and used the hierarchical analysis method in multi-objective decision making to rationalize the data [13]. The data were calculated and analyzed using the hierarchical analysis method in multi-objective decision making, and finally the planning plan of the tourist route with the shortest travel time or the least cost was developed. In this study, the authors introduced multilevel analysis into the design of the tourism route planning scheme, which is a new inspiration for tourism-related research. Secondly, some researchers have also combined the Android system with travel route planning and proposed a personalized travel route recommendation system based on the Android system client and springboot-based back-end; based on this, the attractions in the Beijing region were used as examples, and were finally tested to achieve user login registration and personalized travel route planning recommendation functions [14].

The developing times and the deepening of artificial intelligence research have led to a deeper understanding of artificial intelligence and big data. For this reason, in response to the problems of traditional tourism route planning, such as time-consuming and poor user experience, many researchers have proposed a method to integrate tourism route planning with the ant colony algorithm in artificial intelligence. In this study, the author describes the basic principles of the ant colony algorithm and proposes improvements to the problems of the ant colony algorithm, such as the long time spent, the addition of a real-time pheromone update stage, the centralization of the search range, etc [15]. Finally, the model is applied to the actual case design, and the improved algorithm analysis results in an optimal tourist route that meets the cost and experience [4] goals. In addition, there are also specific cities using the ant colony algorithm to design and study tourism routes [16]. For example, Yaxin Xu added the constraints of touring time and sightseeing value for Fuzhou in the way of travel merchant's problem, and established the mathematical model of touring in the shortest time, so as to realize the study of Fuzhou tourism routes based on the ant colony algorithm.

In addition to the use of the ant colony algorithm to calculate the optimal tourism planning route in the study, some researchers also use the particle swarm algorithm to carry out the shortest tourism route planning; in this planning design, the authors mainly apply the principle of the particle swarm algorithm to the city of Lhasa and find the shortest distance between major popular attractions, so as to enhance the tourist experience, reduce the cost, ensure the tourists' play time and quality, and save costs for tourism companies [17].

The advent of information technology has caused people to think about the value of data, and thus data mining and data analysis have emerged as hot topics for research. At the same time, tourism has become a way for people to relax, enjoy themselves, and broaden their horizons. Although the boom of tourism has brought economic growth, the feeling given to tourists in the planning of tourist routes is not friendly, and there are many problems about youth groups or senior groups of leisure tours but have been taken by the guide to spend [18]. One of the reasons for such problems is the lack of a “human-centered” design concept in the planning and design of tourism routes. For this reason, some scholars have proposed personalized travel route planning recommendations; in order to better exploit and make full use of the database accumulated in the tourism industry, Gong Yuan proposes the use of data mining to design the best travel route planning system, and the combination of hardware and software to achieve the best travel route planning function. In order to highlight the advantages of its route planning, it adds comparison experiments to the experimental process, and the experimental results show that the performance of the best tourism route planning system proposed by the authors is significantly better than the traditional best tourism route planning system [19].

## 2. The Necessity of Tourism Route Planning

China's tourism industry has also entered the stage of comprehensive development of the masses since it entered the comprehensive building of a well-off society, but at the same time China's tourism industry has entered the bottleneck period and some deep-seated contradictions have become more prominent during the epidemic, and these contradictions mainly focus on the dysfunction of the tourism industry structure, the lack of total tourism products, a single type of route, and the industry norms as they are not standardized [20].

### 2.1. Lack of Holistic Planning of Tourism Routes

First of all, Western countries for the planning of tourism routes have formed a specific and more standardized path design process, but China has not formed a set of more standardized design specifications. The reason for this phenomenon is that the line design involves more variables, is a more complex system design process, and the lack of training for this type of talent in China, thus leading to a gap in the market for tourism route planning designers in China [21]. Therefore, the current travel agency's tourist routes are generally undertaken by the relevant departments, and the planning staff in the design of tourist routes, due to the lack of professional knowledge, rely only on the main traffic routes and the scenic nodes as a series of points, resulting in the design of tourist routes in the program of the same without considering the essence of the tourist preferences, a characteristic key factor that should be paid attention to the dynamic changes in the market.

In addition, because the tourist route planning and design of the scenic area does not have a certain unified standard, it results in the design of the tourist route planning program in the short cycle, slow updates, the degree of homogenization, and other problems [22]. Individual units highlight the relationship between the road layout of the scenic spot, ignoring the low carrying capacity caused by the scenic spot route, and the resources have also suffered the same damage. It can be seen that the attention to the tourist route specification scheme design as a whole is a key part in achieving optimal route planning [23].

### 2.2. Not Deep Enough Development of the Product Type of Tourist Routes

**Figure 2:**
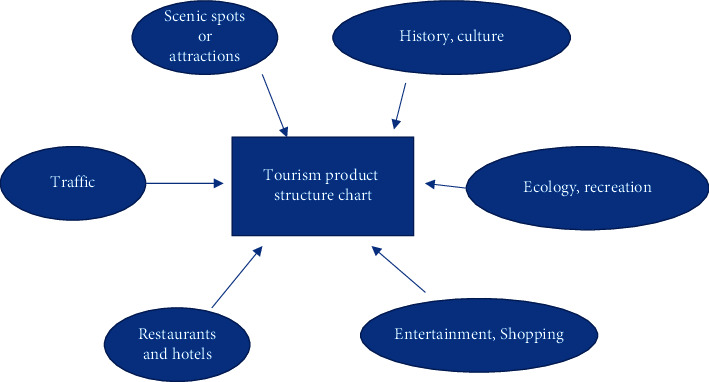
Structure of tourism products.

Nowadays, most of the tourism products in the market are based on sightseeing tourism and vacation tourism, and a single type of tourism gives little space for people to choose, and the design and development of tourism routes is not deep enough. In addition, the route planning scheme is relatively rough and unrefined in design, and most of the routes are not integrated with the local humanities and history. Secondly, some of the smaller travel agencies do not have the concept of developing a variety of tourism products, making it difficult to survive the epidemic and becoming a “stepping stone” for other travel agencies [24]. Some even have thematic tourism routes that do not correspond to the actual phenomenon, which greatly affects the mood and experience of tourists. In summary, the root causes of the above problems are due to the lack of depth in the development of the product range of travel itineraries by travel agencies as shown in Figure 2.

### 2.3. The Design Theme of Tourism Routes Is Not Innovative and Not Compelling Enough

After decades of development of the tourism industry in China, under the guidance of the national tourism department, the characteristics of our tourism resources are actually quite distinct and the number of tourism resources of the same type is high, but the design themes of our tourism routes are not innovative and not compelling enough. The fundamental reason for this problem is that the purpose of today's tourism route design is based on “how to get people to the scenic area at the least cost,” and in the eyes of most tourism route planners, “tourists arrive at the scenic area” is the same as “This equivalence is actually wrong [25].” The essence of such problems is that the designers are not aware of the “contextual design.” For example, in the case of peripatetic tourism, there is a lack of “contextual design,” and most of the tours are designed to allow people to walk around and see the sights while ignoring the local cultural characteristics, history, and customs. In general, the planning and design of the tourist route is not innovative, and does not consider the design concept of the core of the tourists' feelings [26].

### 2.4. The Combination of Design Elements Based on the Tourist Route Is Low

What does the design combination of tourist routes refer to? Is tourism just about going to a place? The answer is not at all. Travel is a way to pack up and go to another place to experience another kind of life, a trip to feel other customs and historical features. Since it is a trip, it inevitably includes food, clothing, housing, transportation, the distance from the place of residence to the scenic spot, the time of the tour, etc [27]. This paper believes that tourism should be the combination of all the above route design elements and the reasonable allocation of resources of all parties; the rationality of tourism route planning also depends on this. However, China's travel agencies in the design of route planning lack of a reasonable configuration of these elements and in the combination of elements of focus on the poor. For example, for Zhuhai–Macau, a two-day tour, the travel agency designed the route mainly as a shopping tour, and focused almost all the time on shopping, and ignored the local attractions of Zhuhai and Macau [28]. Excessive shopping will inevitably lead to dissatisfaction; although the theme is a shopping tour, other attractions and sightseeing time must be involved; otherwise, it will only cause tourists' dissatisfaction with the tour, thus reducing the sense of experience, and losing the original intention to travel is relaxation and happiness [29].

To sum up, through the analysis of relevant literature, coupled with the arrival of the era of artificial intelligence and big data, coupled with the overall development of tourism, there are many challenges in the development direction of tourism under the epidemic. Tourism route planning solutions are also emerging in an endless stream, whether in terms of improvement or in the application level, they are very effective. Among them, the clustering algorithm is more intuitive than the traditional algorithm. Therefore, this paper proposes the use of cluster analysis algorithms to analyze tourism route planning schemes and incorporate the concept of “wisdom” to make tourism route planning more human-like in thinking. The next paper will analyze the classification and basic principles of cluster analysis in order to select the appropriate cluster analysis algorithm to find the best tourism route planning plan.

## 3. Clustering Analysis Algorithm

Clustering analysis algorithm is an important research topic based on data mining. Data mining refers to the ability to mine and discover valuable, meaningful, and implicit potential knowledge or rules from a large amount of data in order to give decision support to the problems provided by users [30].

In data mining, the main algorithms are the association rule analysis algorithm, decision tree, classification pattern, clustering pattern analysis, and nowadays the widely researched neural network algorithm. Among them, the clustering analysis algorithm is a very effective unsupervised machine learning algorithm for the classification of certain [8]problems. Clustering is different from classification in that classification is a way of classifying things by type, nature, or rank, while clustering is a way of partitioning data in a dataset into different clusters or classes according to a specific criterion or rule, and making the similarity or correlation of data objects in the same class or cluster as large as possible. Essentially, data objects with the same properties are grouped in the same “group” (cluster) under a certain criterion, and those with more different properties are placed in another group. In short, clustering itself is an unsupervised learning method that does not care about the labels of the data, but only whether the aggregated data are in the same cluster or the same category.

**Figure 3:**
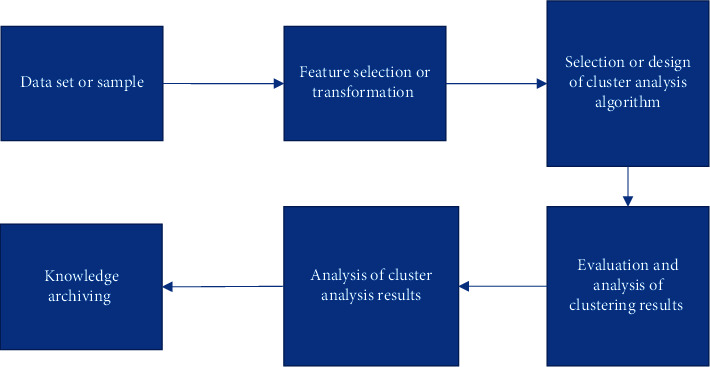
The process of cluster analysis.

Cluster analysis is a rigorous mathematical process; the algorithm has been extensively studied for many years since it was proposed in 1984, and has evolved from the initial four processes to the following six basic steps as shown in Figure 3.

The first step is to determine the dataset or sample, followed by the preprocessing of the dataset or sample; the second step is the selection or transformation of features, which refers to the selection of suitable variables, and the selection of variables is related to the efficiency and correctness of the subsequent algorithm; thus, this is an important and critical step; after the selection of suitable variables, the next step is the scaling of data, the search for outliers, etc. The next step is to select a suitable clustering algorithm or to improve the clustering analysis algorithm, which will be discussed in Section 3.1; the next step is to confirm the number of classes, i.e., to evaluate and analyze the clustering results, and finally to visualize the clustering results, so as to realize the verification of the results.

### 3.1. Basic Methods of Data Clustering Classification

Nowadays, mainstream data-based clustering analysis algorithms can be classified into the following categories: hierarchical methods, division methods, density-based clustering methods, new methods, etc. Next, the following different types of clustering algorithms are analyzed and compared, so as to select the appropriate cluster analysis algorithm for the design of the tourism route planning scheme.

**Figure 4:**
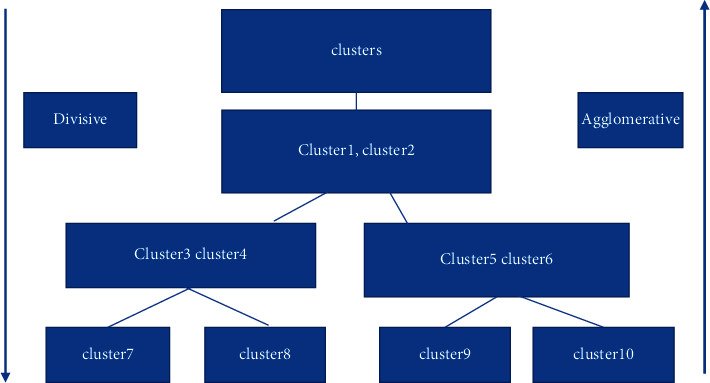
Comparison of agglomerative hierarchical cluster analysis and divisive hierarchical cluster analysis algorithms.

First is the hierarchical cluster analysis algorithm. The hierarchical cluster analysis algorithm is firstly “hierarchical,” i.e., the dataset or sample is divided into different levels of clusters, and the clusters generated by the later layer are based on the results of the previous layer, and the hierarchical cluster analysis algorithm generally has the following two kinds: the first one is agglomerative hierarchical clustering, followed by divisive hierarchical clustering. In agglomerative hierarchical clustering, each selected object starts with a cluster, i.e., clusters, and each time the two closest clusters are merged to generate a new cluster according to certain criteria or standards, and the process is cycled until all clusters are merged into one large cluster. It should be noted that agglomerative hierarchical clustering is suitable for mixed data types, with better noise immunity and arbitrary cluster shapes, but its algorithm is slow; for divisive hierarchical clustering, also known as the top-down (i.e., top-down) hierarchical clustering analysis algorithm, and the above-mentioned hierarchical clustering algorithm, what is different from the above hierarchical clustering is that the algorithm puts all the datasets or samples into one big class, i.e., clusters, and each time divides a certain clusters into multiple clusters according to certain criteria or guidelines, and keeps looping this process until all the clusters can no longer be classified, i.e., at this time all the clusters are a single cluster as shown in Figure 4.

**Figure 5:**
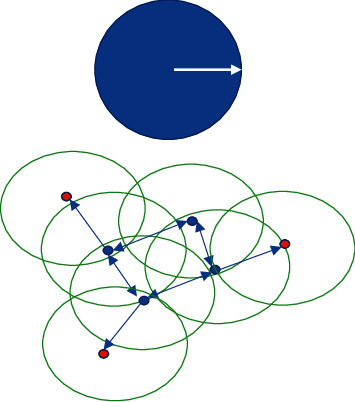
The basic principle of the DBSCAN algorithm.

Next is the density-based cluster analysis algorithm. In this cluster analysis algorithm, density is mainly used instead of similarity, and the density of the distribution of data or sample objects as a starting point; the density cluster analysis algorithm is applied on this basis to select regions large enough to be connected such that clusters of various shapes can be composed in the data. One of the most classic density-based cluster analysis algorithms is the DBSCAN algorithm proposed by Ester et al. [10]. In the DBSCAN algorithm, two parameters *M* and *μ* need to be defined, where *M* denotes the neighborhood density threshold of the clusters and *μ* denotes the neighborhood radius that defines the density. The idea of the algorithm is to first discover the points with higher density, and then connect the points with similar densities according to a certain minimum threshold specified by the standard that the objects contained in the neighborhood of a given radius cannot be less, and gradually connect them together, and then generate various [11] clusters. The principle of one of the algorithm implementations is as shown in Figure 5.

As can be seen in Figure 5, the circle above the blue color represents the base cluster and its radius represents the neighborhood radius of the density *μ*. In this algorithm, first of all, for each data object as the center of the circle, draw a circle with the neighborhood as the radius; if a circle has as many points in it, then this number is the density value of that point. Then a suitable density threshold minpts is selected and the density threshold is used as the reference; if the points within the circle are higher than the density threshold, they are classified as high-density points; conversely, if they are lower than the density threshold, they are treated as low-density points. If two or more high-density points are both in the same circle, the two points are connected with two-way arrows. If there are low-density points within a circle with high-density points, they are also connected, eventually forming a cluster. As shown in the figure, blue points indicate high-density points, red points indicate boundary points, and anomalous points do not appear in this cluster. All the blue and yellow points are connected together to form a cluster.

Based on the above analysis, it is clear that the DBSCAN algorithm has the following characteristics:

1. The division of cluster classes is based on the parameter *μ* and the cluster density threshold *M* value, and both the parameters are considered to be set
2. It is sensitive to the selection of initial values, but insensitive to noise points and has certain noise immunity
3. It is not necessary to set the number of clusters in advance
4. The clustering effect is poor for data with uneven density, and it is only applicable to the aggregation of more concentrated data or samples

The last is the analysis of the division-based clustering analysis algorithm. Delineation clustering analysis is the most common and basic clustering algorithm. The basic idea of delineation clustering is to divide a dataset or sample that needs to be divided or aggregated into *k* [12] clusters. In the partitioned clustering analysis algorithm, the *k* value, i.e., the number of cluster centers or clusters, is specified in advance, and then the data or samples are initially clustered, and after repeated iterations, the final goal of “the points within the cluster are close enough and the points between the cluster and the cluster are far enough” is achieved. The clustering result is obtained when the goal of “the points within the cluster are close enough and the points between the cluster and the cluster are far enough” is reached, i.e., the optimal solution state is reached or the clustering can no longer be performed. The common divisional clustering analysis algorithms include k-means clustering algorithm, its variant k-means++ clustering algorithm, as well as bi-kmeans clustering algorithm and kernel k-means clustering algorithm. The next section focuses on the most classical k-means clustering algorithm.

### 3.2. *k*-Means Clustering Analysis Algorithm

Among the clustering analysis algorithms, the main research is mainly in distance-based clustering analysis. The typical representative of this kind of cluster analysis is the k-means algorithm. The *k*-means clustering analysis algorithm is a dynamic clustering algorithm, in which the initial value of *k* is given, and the samples or datasets to be measured are divided into *k* classes, and then the distance from the sample to the center of the cluster is the smallest or shortest sum of squares for the purpose of classifying the samples or datasets. In the k-means clustering algorithm, firstly, *k* is used as a parameter criterion as the size of the number of clusters to divide *n* data objects or samples into *k* clusters, such that the data objects or samples or points within a cluster have a high similarity (which can also be called the shortest distance) and a high dissimilarity between clusters (which can also be called a long distance beyond the set distance threshold).

**Figure 6:**
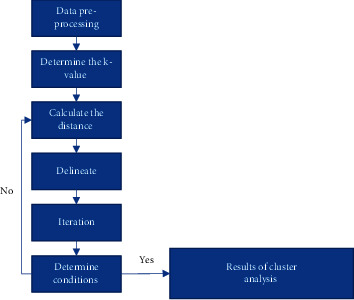
Classical k-means clustering analysis algorithm flow.

The process of the classical k-means cluster analysis algorithm is as shown in Figure 6.

As can be seen from Figure 6, for the classical k-means cluster analysis algorithm, the first step is to determine the number of categories and the center of mass after preprocessing the data, and then to divide and label the clusters by calculating the Euclidean distance of the data objects or cluster centers, in which the data points are assigned to the nearest clusters; after this step is completed, the iterative process is entered, and the mean value of each cluster is recalculated for each of the previously divided cluster. After this step, the iterative process is carried out, and the mean value of each cluster is recalculated and used as the center of mass, i.e., the center of clusters. The above process is repeated for all data objects or samples until all clusters can no longer be divided, and the final result of cluster analysis is obtained.

In summary, the *k*-means cluster analysis algorithm has the following characteristics:

1. Need to consider setting the k-value
2. Dependent on and sensitive to the initial center of mass (clustering center)
3. It is more sensitive to abnormal data or samples

## 4. Intelligent Tourism Route Planning Scheme Design

### 4.1. Algorithm of Smart Tourism Route Planning

After the analysis in Section 3, three classification methods of cluster analysis algorithms are commonly used, which are density-based cluster analysis algorithm, division-based cluster analysis algorithm, and hierarchical cluster analysis algorithm. There are advantages and disadvantages in each of these three algorithms.

For the density-based cluster analysis algorithm, its advantage is that it can cluster dense datasets of any shape, but unlike the k-means cluster analysis algorithm, the density cluster analysis algorithm is not sensitive to the outliers in the dataset, and it also has the ability to find outliers while clustering. If the density of the dataset or sample is not uniform or the density of clusters between clusters differs greatly, it will lead to poorer clustering results; more importantly, for the case of a large number of datasets or sample sets, the density clustering analysis algorithm clustering process takes longer and the convergence time is slower; the adjustment parameters are mainly for the neighborhood density threshold minpts and the distance threshold *μ* joint. This is more complicated than the traditional *k*-means clustering algorithm, and different combinations of parameters have a greater impact on the final clustering results.

For the hierarchical cluster analysis algorithm, its advantage is that the definition of the prescribed similarity is easier, the selection of the distance is not difficult, and there is no need to develop the *k*-value and the number of clusters in advance; due to the nature of the algorithm, it itself can also discover the hierarchical relationship of the classes and the diversity of the shapes of the clusters; however, compared to the k-means cluster analysis algorithm, the complexity of the calculation is too high, and it also generates singular values, which has a great impact on the quality of the clustering. However, compared to the k-means clustering analysis algorithm, the computational complexity is too high, and it also generates singular values, which has a great impact on the quality of clustering results, and the shape of the clusters is likely to be clustered into chains.

Therefore, through the above analysis, for the optimal route selection problem in tourism route planning, compared with the other two types of cluster analysis algorithms, the k-means cluster analysis algorithm has the advantages of simplicity, easy to understand and implement, and low time complexity, but the traditional k-means algorithm has the sensitivity to the initial value and some excessive outliers will bring great influence to the clustering results; thus, this paper decided to improve the k-means cluster analysis algorithm, and the improved cluster analysis algorithm is used to make the optimal planning of the tourist routes in a city, and to propose the design of an intelligent tourist route planning scheme.

### 4.2. Scheme Design and Result Analysis

**Figure 7:**
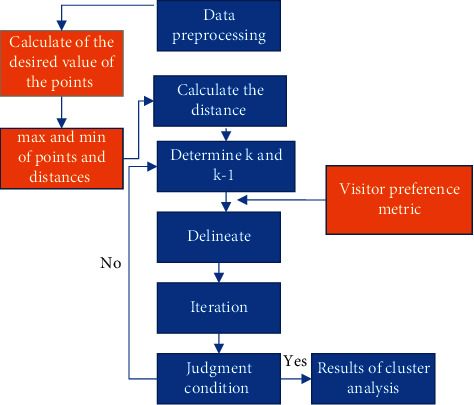
Improved *k*-means clustering analysis algorithm flow.

For the traditional k-means cluster analysis algorithm, its biggest drawback lies in determining the k-value in advance, i.e., *k* points have to be selected as the center of the initial clustering beforehand, and then the clustering center can be updated iteratively on this [9]basis. Therefore, the traditional k-means clustering analysis algorithm is very dependent on the initial value, and once the initial value is not properly selected, it will lead to poor quality of the final clustering results. Therefore, in order to solve the problem that the traditional *k*-means clustering analysis algorithm is very dependent on the initial value, this paper proposes to find the expected value of all points of the data volume or sample after data preprocessing, and then select the largest and smallest points as the two clustering centers according to the Euclidean distance between the expected value and the points, i.e., class *k* and class *k* − 1; then, repeat the above process until the clustering is completed as shown in Figure 7.

**Figure 8:**
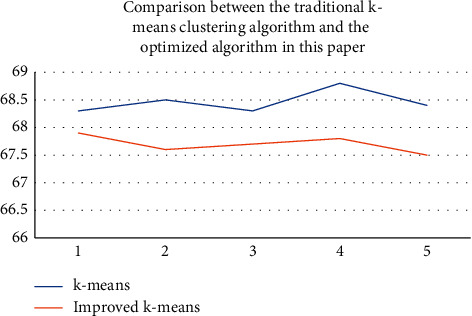
Comparison of the traditional *k*-means clustering analysis algorithm and the improved algorithm in this paper for the optimization of smart tourism road route planning.

For the planning of smart tourism routes, this paper first constructs a dataset of *m* classes representing the IDs of *m* attractions in a city, where each ID has its own attributes, followed by finding the expected value for all the distances of the *m* attractions, and then calculating the Euclidean distance between the attractions according to the calculated expected value, selecting the maximum and minimum values of the Euclidean distance as *k* classes and *k* − 1 classes; at the same time, adding the tourists' preference metric, it is then divided and iterated, and the above process is repeated continuously, in addition to averaging between satisfying tourists' preferences and distances; routes below the average will be directly excluded, forming the optimal tourist routes in turn. At last, the results of this clustering analysis are judged by the sum of the distances between the traditional k-means clustering and the improved k-means clustering analysis algorithms of this paper as shown in Figure 8.

**Figure 9:**
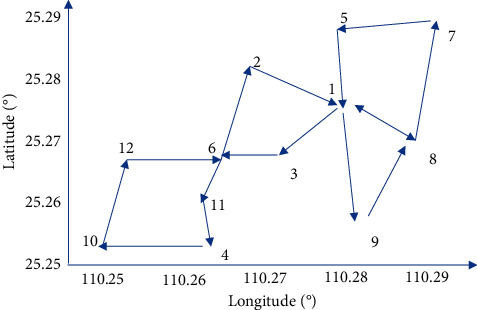
Intelligent tourism route planning results based on the improved k-means cluster analysis.

Figure 8 shows that the sum of distances is used as the criterion for judging the clustering results, followed by the horizontal coordinate indicating the number of clusters and the vertical coordinate indicating the sum of distances, where the smaller the sum of distances, the better the clustering results. From the results in Figure 8, it can be seen that the improved k-means algorithm is significantly better than the traditional k-means clustering algorithm in terms of clustering results. Finally, after adding the tourist preference metric as a reference factor, the smart tourism route planning based on cluster analysis for a city in this paper is as shown in Figure 9.

From the experimental results in Figure 9, it can be seen that the intelligent tourism route planning for a city based on the improved k-means cluster analysis algorithm is reasonable, and the system fully takes into account the interest measure of tourists' preferences, which has a reference value for both tourism agencies and tourists.

## 5. Conclusion

This paper analyzed the development of the tourism industry in today's epidemic state, the problems and current situation of tourism route planning, and designed a smart tourism route planning scheme based on the cluster analysis algorithm in order to improve the rationality and achieve optimization of tourism route planning; in order to solve the problem of high dependence of the traditional k-means cluster analysis algorithm on the initial value, the improvement of the algorithm was proposed. In order to solve the problem of high dependence of the traditional *k*-means clustering analysis algorithm on initial values, the algorithm is improved and successfully applied to the route planning of a tourist city while introducing the tourist favorite interest metric, and the experiment verifies that the scheme achieves good results.

However, there are still some shortcomings in the design of the scheme, such as an optimal intelligent tourism route planning scheme should be complete, with strong coverage and meet the individual needs of tourists in various aspects such as clothing, food, accommodation, and transportation; this paper only aims at optimizing the route with the shortest route and considering tourists' preferences, which has some reference value, but more considerations can be added in the subsequent research to make the optimal tourism route/scheme. This paper only aims at optimizing the routes with the shortest routes and considering tourists' preferences.

## Data Availability

The dataset can be accessed upon request to the author.
